# Association of COVID-19 infection with welfare recipients in Japan: a retrospective cohort study

**DOI:** 10.1186/s12875-026-03504-1

**Published:** 2026-07-31

**Authors:** Shuhei Yoshida, Daisuke Miyamori, Masanori Ito

**Affiliations:** Department of General Internal Medicine, Hiroshima University Hospital, 1-2-3 Kasumi, Minami-ku, Hiroshima-shi, Hiroshima-ken 734-8551 Japan

**Keywords:** Welfare recipients, Public assistance, COVID-19, Social inequity

## Abstract

**Background:**

The COVID-19 pandemic has exacerbated poverty among vulnerable populations worldwide. However, no study has clarified the extent to which COVID-19 infection increases the likelihood of becoming a welfare recipient in Japan. Therefore, we examined the association between diagnosed/confirmed COVID-19 infection and the probability of receiving public assistance.

**Methods:**

This retrospective cohort study used data from the National Database of five Japanese prefectures. We conducted multivariate analyses using Generalized Estimating Equations (GEEs) as the primary analytic approach. The outcome variable was whether a participant received public assistance. The main exposure variable was diagnosed/confirmed COVID-19 infection status, and covariates included age, sex, and quarter. Additionally, we used a General Linear Model (GLM) to assess the risk of requiring public assistance within three months after infection among individuals with diagnosed/confirmed COVID-19 as a secondary analysis. Explanatory variables in this analysis included age, sex, prescriptions, and utilization of care services in 2019.

**Results:**

The total sample comprised 16,712,657 participants (7,247,496 with diagnosed/confirmed COVID-19 and 9,465,161 without diagnosed/confirmed COVID-19). The GEE analysis showed that the Incidence Rate Ratio (IRR) was highest in the month of diagnosed/confirmed COVID-19 infection (IRR: 2.57, 95% CI: 2.54–2.59, *p* < 0.001) and subsequently showed a gradual decline. The GLM analysis indicated that not using outpatient or inpatient care services in 2019 was associated with the highest risk of receiving public assistance (IRR: 25.3, 95% CI: 23.3–27.5).

**Conclusions:**

The diagnosed/confirmed COVID-19 infection was associated with an increased likelihood of receiving public assistance. Furthermore, individuals who had not utilized hospital or clinic services in 2019 were at the highest risk of becoming welfare recipients compared with other individuals.

**Supplementary Information:**

The online version contains supplementary material available at https://doi.org/10.1186/s12875-026-03504-1.

## Introduction

Due to the COVID-19 pandemic, the global population living in extreme poverty increased by an estimated 90 million in 2020, marking the largest rise since 1990 [1]. Individuals experiencing poverty or social marginalization face a higher risk of infection and disproportionately negative outcomes because of unsafe living conditions and a greater prevalence of comorbid medical conditions [2]. Although previous studies have demonstrated that pandemics exacerbate poverty and that people living in poverty are at increased risk of infection, the risk of falling into poverty as a direct consequence of COVID-19 infection has not yet been investigated.

Japan’s public assistance system is designed to ensure a minimum standard of living for individuals and households who, due to circumstances such as unemployment, illness, disability, or unexpected accidents, are unable to maintain basic living standards even after exhausting all available assets and support [3]. The system provides cash benefits and in-kind assistance to cover essential needs such as living expenses, housing costs, medical care, and education. Eligibility is determined through a rigorous assessment of assets and income [4]. According to the Ministry of Health, Labour and Welfare, the relative poverty rate is 15.4%, ranking fifth among countries in the Organization for Economic Co-operation and Development report [5, 6]. However, in practice, only approximately 1.6% of the Japanese population received public assistance in 2023, with the majority being elderly (55.3%) or households with disabilities (25.0%) [7]. Individuals receiving public assistance in Japan face numerous health challenges and are at a disadvantage compared with the general population [8]. Previous studies using large-scale cohorts have demonstrated that welfare recipients (WRs) face significantly higher risks of all-cause mortality, cardiovascular disease, and suicide than the general population [8–11]. Furthermore, mental health conditions, particularly depression and schizophrenia, have been identified as both critical predictors of entering the welfare system and major barriers to social reintegration. While previous epidemiological research in Japan has extensively documented the high disease burden and mortality among existing WRs or the impact of the pandemic on the quality of life of low-income populations, longitudinal evidence tracking the transition to welfare receipt following acute health shocks, such as the COVID-19 infection, remains scarce [8, 12, 13]. Furthermore, although the association between poverty and healthcare underutilization has been established, the role of pre-pandemic healthcare engagement in predicting future socioeconomic vulnerability during disaster has not been quantified. Therefore, in this study, we first examined the association between COVID-19 infection and the risk of receiving public assistance. Furthermore, we investigated differences in medical care between individuals who required public assistance after contracting COVID-19 and those who did not. By evaluating factors, such as treatment for chronic diseases, polypharmacy, provision of home medical care, admission to care facilities, and treatment for psychiatric disorders, we aimed to quantitatively identify high-risk individuals in routine clinical practice. This study sought to clarify these factors and contribute to preparedness for future pandemics or disaster situations.

## Methods

### Study design

This study employed a retrospective cohort design.

Health insurance system and public assistance system in Japan.

Japan implemented a universal health insurance system in 1961. For most insured individuals, the copayment rate is set at 30%. Individuals aged 75 years and older with incomes below the national average pay only 10% as a copayment. Those aged 70–74 years with below-average incomes, as well as children under six years of age, are required to pay 20%. Numerous prefectures and municipalities have established their own medical expense subsidy programs to partially or fully cover the copayment burden for children. If a monthly copayment exceeds a threshold determined by the insurer, the high-cost medical expense benefit covers the amount exceeding this limit.

WRs receiving public assistance are exempt from copayments when using medical services. They present medical vouchers provided by municipal offices to medical institutions at the time of care. The medical institution subsequently bills the municipal welfare office, which directly reimburses the institution for the medical expenses. Copayment assistance is not provided unless a medical facility is used. The public assistance in Japan is determined based on the household units rather than individuals. All family members are collectively certified as recipients when the household’s total income falls below the eligibility threshold, regardless of age.

### Data source

This study utilized the National Database of Health Insurance Claims (NDB), an administrative claims database in Japan. Although the NDB includes nearly all medical service data in the country, it excludes individuals covered by workers’ accident insurance or those insured through automobile liability or workers’ compensation insurance. The proportion of excluded patients was less than 2%. Authorization to use a subset of the NDB as a special extraction sample for this specific research project was obtained from the Ministry of Health, Labour and Welfare (approval no. 2502). The scope of this authorized sample included the five prefectures. Although this represents a subset of the national data, the combined population of these prefectures (approximately 20.5 million) accounts for approximately 16% of the total Japanese population, ensuring sufficient scale and statistical power for longitudinal analysis.

### Setting and participants

The study was conducted in five prefectures—Hiroshima, Kyoto, Osaka, Okayama, and Hyogo—covering a population of approximately 20.5 million people, or about 16% of Japan’s total population. The NDB does not contain participants’ residential addresses but includes the addresses of medical institutions. Therefore, the study area was defined based on the location of the medical institution that generated the claim. Participants were individuals recorded in the NDB from January 2020 to March 2022 in the five prefectures. Because the NDB does not record patient deaths, we excluded individuals who had no medical claims during the final three months of the observation period. To focus on new cases of public assistance, we also excluded participants who were WRs in 2019.

### Variables

Certification as a WR was the outcome variable. Each month, we determined whether a participant was a WR. The presence of a specific public expense number on medical claims was used to ascertain exposure status. During the study period, COVID-19 was a notifiable disease in Japan, and all medical expenses for diagnosed patients were fully covered by public funds. These payments are recorded in the NDB with a unique identifier (public expense number). We identified participants with diagnosed/confirmed COVID-19 infection by detecting this number in the monthly claims data. The “month of infection” was defined as the calendar month in which the claim containing this public expense number first appeared.

The primary exposure variable was diagnosed/confirmed COVID-19 status. The diagnosed/confirmed COVID-19 infection was identified at both the individual and monthly levels. The diagnosed/confirmed COVID-19 status was categorized as uninfected, month of infection, 1–3 months after infection, 4–6 months after infection, 7–9 months after infection, 10–12 months after infection, and more than one year after infection. When reinfection was identified, the time since infection was recalculated.

Age and sex were extracted from data sources. In our analysis, age was designated as a categorical variable to avoid assuming a linear relationship across the lifespan. In addition to age and sex, the calendar quarter for each observation was determined. Specifically, the observation period was divided into three-month intervals (e.g., 2020 Q1, 2020 Q2) starting from January 2020. To account for time-varying social conditions and macro-level influences during the pandemic, the calendar quarter was included as a covariate in the GEE models. This allowed us to adjust for period-specific confounding factors that could influence the likelihood of receiving public assistance, including the introduction of COVID-19 vaccines, shifts in dominant viral variants, and the implementation of various social protection policies such as special cash payments (emergency cash transfers) and employment adjustment subsidies. By incorporating this temporal factor, we aimed to isolate the association between individual-level COVID-19 infection and the risk of welfare receipt from the broader socioeconomic impact of the pandemic.

The secondary outcome analysis was limited to participants diagnosed with COVID-19 infection, and their use of specific medications and healthcare facilities in 2019 was examined. The medications included hypnotic and sedative agents, antidepressants, psychotropic agents, antihypertensive agents, lipid-lowering agents, antidiabetic agents, and Chinese herbal medicines. This classification is based on a 12-digit alphanumeric code maintained by the Ministry of Health, Labour and Welfare and published by the Medical Affairs Division of the Health Insurance Bureau [14]. Healthcare utilization was categorized as outpatient and inpatient care, outpatient care only, inpatient care only, or no care.

### Statistical analysis

#### Primary analysis

Association of the diagnosed/confirmed COVID-19 infection on receipt of public assistance.

We described the baseline characteristics of participants with and without diagnosed/confirmed COVID-19 infection. The chi-square test was used for categorical variables. For ordinal and non-normally distributed continuous variables, we performed the Wilcoxon rank-sum test.

We conducted multivariate analyses using Generalized Estimating Equations (GEEs) with monthly panel data for each participant. Our primary analysis utilized longitudinal panel data with a modified Poisson regression within the GEE framework to estimate the incidence rate ratio (IRR). The outcome variable was whether a participant received public assistance. The main exposure variable was diagnosed/confirmed COVID-19 status, and covariates included age, sex, and quarter. The GEE models were specified with a Poisson distribution, log link function, and robust error variance. We then assessed the statistical significance of interaction terms between age and diagnosed/confirmed COVID-19 status, and between sex and diagnosed/confirmed COVID-19 status. Based on these results, we conducted subgroup analyses stratified by age and sex. In these analyses using the GEE, participants were not censored after the first occurrence of welfare receipt to continuously assess the probability of being a welfare recipient relative to the time elapsed since COVID-19 diagnosis. For participants with multiple infections, the diagnosed/confirmed COVID-19 status was updated based on the month of the most recent confirmed diagnosis.

#### Secondary analysis

In addition to estimating the risk of the primary outcome in the overall population, we assessed the risk of receiving public assistance among participants with diagnosed/confirmed COVID-19. Healthcare providers frequently encounter patients for the first time during diagnosed/confirmed COVID-19 infection, which often leads to subsequent medical care. By evaluating these patients, we aimed to quantify the risk from a clinical perspective.

The secondary analysis utilized a cross-sectional data design with a modified Poisson regression in a General Linear Model (GLM) framework. We applied a GLM with a Poisson distribution, log link function, and robust error variance. This model was also performed to estimate the IRR. The outcome variable was whether a participant received public assistance within three months after diagnosed/confirmed COVID-19 infection. Explanatory variables included age, sex, prescriptions, and utilization of care services in 2019. We then assessed the statistical significance of interaction terms between age and utilization of care services and between sex and utilization of care services in 2019. Based on these results, we conducted subgroup analyses stratified by age and sex.

To ensure the robustness of our findings, a sensitivity analysis was applied that excluded individuals < 20 years of age because the drivers of public assistance entry for minors may differ from those for adults. Since the exclusion of this group did not significantly alter the estimates or the primary conclusions through the primary and secondary analyses, the all-age model was maintained to reflect the socioeconomic impact of COVID-19 infection on the entire household unit.

All statistical analyses were performed using STATA/SE version 18 (StataCorp, 2023).

## Results

Table 1 presents the characteristics of the study participants. The total sample comprised 16,712,657 individuals (7,247,496 with diagnosed/confirmed COVID-19 and 9,465,161 without diagnosed/confirmed COVID-19).

**Table 1:** Characteristics of study participants

Characteristic		Participants with diagnosed/confirmed COVID-19 (*n* = 7,247,496)	Participants without diagnosed/confirmed COVID-19 (*n* = 9,465,161)	*p* value
Age category, n (%):	0–19	1,935,616 (26.7)	1,271,448 (13.4)	< 0.001^*^
	20–39	1,777,552 (24.5)	1,701,351 (18.0)	
	40–59	1,607,027 (22.2)	2,578,316 (27.2)	
	60–79	1,347,534 (18.6)	2,989,244 (31.6)	
	≥ 80	579,767 (8.0)	924,802 (9.8)	
Sex, n (%):	Male	3,374,355 (46.6)	4,201,704 (44.4)	< 0.001^*^
	Female	3,873,141 (53.4)	5,263,457 (55.6)	
Welfare recipient, n (%):		61,493 (0.9)	84,879 (0.9)	< 0.001^*^
Total months receiving public assistance among welfare recipients, median (interquartile range):		8 (3–15)	5 (2–13)	< 0.001^**^

^*^Chi-square test

^**^Wilcoxon rank-sum test

Table 2 presents the results of the GEE analysis examining the association between receipt of public assistance and COVID-19 status. The IRR was highest during the month of diagnosed/confirmed COVID-19 infection (IRR = 2.57, 95% CI: 2.54–2.59, *p* < 0.001) and subsequently demonstrated a gradual decline.

**Table 2:** Results of generalized estimating equations examining the association between receipt of public assistance and COVID-19 status

Variable		IRR	95% CI		*P* value
			Under	Upper	
Age category	0–19	Reference			
	20–39	1.36	1.31	1.42	< 0.001
	40–59	2.76	2.66	2.87	< 0.001
	60–79	4.81	4.63	4.99	< 0.001
	≥ 80	4.81	4.63	5.01	< 0.001
Sex	Male	Reference			
	Female	0.84	0.82	0.85	< 0.001
Diagnosed/confirmed COVID-19 status	Uninfected	Reference			
	Month of infection	2.57	2.54	2.59	< 0.001
	1–3 months after infection	1.53	1.52	1.55	< 0.001
	4–6 months after infection	1.32	1.30	1.34	< 0.001
	7–9 months after infection	1.22	1.20	1.24	< 0.001
	10–12 months after infection	1.15	1.12	1.18	< 0.001
	> 1 year after infection	1.05	1.02	1.09	< 0.001
Calendar Quarter	2020 Q1	Reference			
	2020 Q 2	1.70	1.68	1.73	< 0.001
	2020 Q 3	2.42	2.38	2.46	< 0.001
	2020 Q 4	3.04	2.99	3.09	< 0.001
	2021 Q 1	3.44	3.39	3.50	< 0.001
	2021 Q 2	4.00	3.93	4.07	< 0.001
	2021 Q 3	4.39	4.32	4.47	< 0.001
	2021 Q 4	4.95	4.86	5.03	< 0.001
	2022 Q 1	4.99	4.90	5.07	< 0.001
	2022 Q 2	5.44	5.35	5.53	< 0.001
	2022 Q 3	5.49	5.40	5.59	< 0.001
	2022 Q 4	5.77	5.67	5.87	< 0.001

**Fig. 1:**
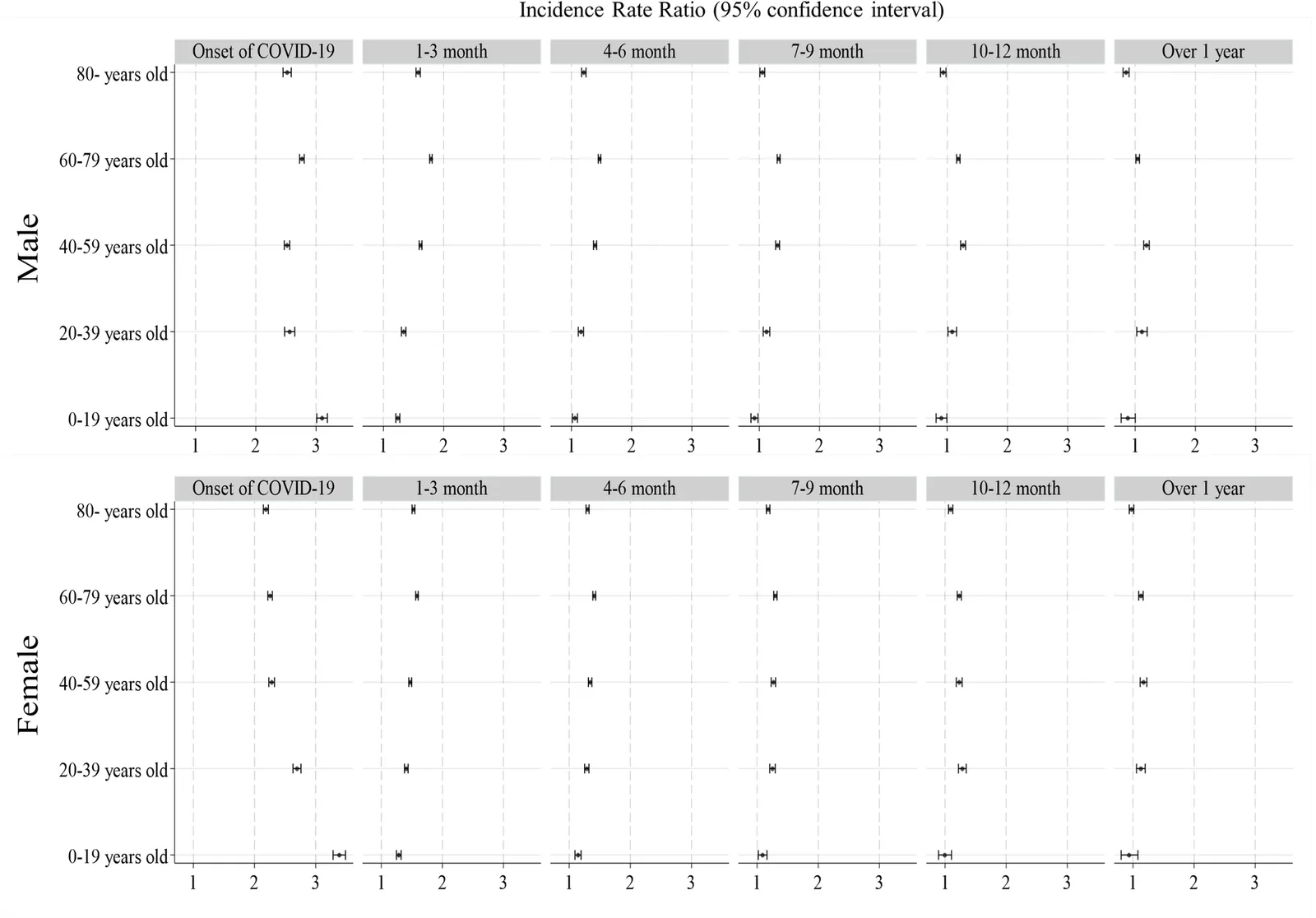
Results of subgroup analyses examining receipt of public assistance by age category and sex. Footnote: Incidence rate ratios (IRRs) and 95% confidence intervals (CIs) were estimated using Generalized Estimating Equations (GEEs), adjusted for observation quarter. The uninfected group serves as the reference for each subgroup. Abbreviations: IRR, incidence rate ratio; CI, confidence interval

Figure 1 presents the results of subgroup analyses stratified by age category and sex, after we assessed the statistical significance of interaction terms (Supplementary Table 1). Younger age groups exhibited a higher risk of receiving public assistance immediately after diagnosed/confirmed infection, but this risk declined rapidly thereafter. In contrast, older age groups demonstrated a more persistent risk, although the risk did not increase immediately after diagnosed/confirmed infection as observed in the younger groups.

Table 3 presents the characteristics of participants infected with diagnosed/confirmed COVID-19. The highest proportion of WRs was observed among participants who did not use either inpatient or outpatient care.

**Table 3:** Characteristics of participants diagnosed/confirmed as COVID-19 by utilization of care services in 2019

Characteristic		Utilization of care services in 2019							
		Outpatient and inpatient care (*n*=728,732)		Outpatient care only (*n*=5,596,633)		Inpatient care only (*n*=21,025)		No care (*n*=901,106)	
		*n*	%	*n*	%	*n*	%	*n*	%
Age category, *n* (%)	0–19	107,775	13.8	1,507,652	26.9	4,763	22.7	315,426	35.0
	20–39	109,367	14.0	1,350,640	24.1	1,880	8.9	315,665	35.0
	40–59	104,337	13.3	1,315,619	23.5	4,023	19.1	183,048	20.3
	60–79	240,496	30.7	1,029,743	18.4	6,653	31.6	70,642	7.8
	≥ 80	166,757	21.3	392,979	7.0	3,706	17.6	16,325	1.8
Sex, *n* (%)	Male	332,307	42.5	2,535,106	45.3	9,842	46.8	497,100	55.2
	Female	396,425	50.6	3,061,527	54.7	11,183	53.2	404,006	44.8
Prescriptions, *n* (%)	Hypnotic and sedative agents, antidepressants	114,846	14.7	393,053	7.0	42	0.2	734	0.1
	Psychotropic agents	93,352	11.9	350,986	6.3	36	0.2	606	0.1
	Antihypertensive agents	175,624	22.4	533,095	9.5	62	0.3	1,087	0.1
	Lipid-lowering agents	138,215	17.7	493,232	8.8	29	0.1	846	0.1
	Antidiabetic agents	79,158	10.1	217,814	3.9	30	0.1	462	0.1
	Chinese herbal medicines	176,760	22.6	945,539	16.9	36	0.2	900	0.1
Receiving public assistance within 3 months after diagnosed/confirmed COVID-19 infection, *n* (%)		885	0.1	4,197	0.1	112	0.5	9,605	1.1

Individuals classified as “Inpatient care only” or “No care” did not receive outpatient care within the subject area. Their identified prescriptions were the cases in which they utilized outpatient services at medical institutions outside the subject area and received prescriptions at pharmacies within the subject area

Table 4 presents the results of the GLM examining the association between receipt of public assistance and participant characteristics among individuals diagnosed/confirmed as COVID-19. The risk of receiving public assistance was higher among those prescribed certain medications, including hypnotic and sedative agents, antidepressants (IRR = 1.41, 95% CI: 1.31–1.51), psychotropic agents (IRR = 2.15, 95% CI: 2.00–2.31), and antidiabetic agents (IRR = 1.16, 95% CI: 1.05–1.27). Conversely, the risk was lower among participants prescribed antihypertensive agents (IRR = 0.82, 95% CI: 0.77–0.88), lipid-lowering agents (IRR = 0.64, 95% CI: 0.59–0.69), and Chinese herbal medicines (IRR = 0.71, 95% CI: 0.66–0.76). Notably, participants who had not used either outpatient or inpatient care in 2019 had the highest risk of receiving public assistance (IRR = 25.3, 95% CI: 23.3–27.5).

**Table 4:** Results of general linear model examining the association between receipt of public assistance and participant characteristics among individuals diagnosed/confirmed as COVID-19

Variable		IRR	95% CI		*P* value
			Under	Upper	
Age category	0–19	Reference			
	20–39	0.99	0.93	1.06	0.86
	40–59	2.74	2.59	2.90	< 0.001
	60–79	8.84	8.38	9.34	< 0.001
	≥ 80	11.16	10.43	11.95	< 0.001
Sex, *n* (%)	Male	Reference			
	Female	0.86	0.83	0.89	< 0.001
Prescription	Hypnotic and sedative agents, antidepressants	1.41	1.31	1.51	< 0.001
	Psychotropic agents	2.15	2.00	2.31	< 0.001
	Antihypertensive agents	0.82	0.77	0.88	< 0.001
	Lipid-lowering agents	0.64	0.59	0.69	< 0.001
	Antidiabetic agents	1.16	1.05	1.27	0.002
	Chinese herbal medicines	0.71	0.66	0.76	< 0.001
Utilization of care services in 2019	Outpatient and inpatient care	Reference			
	Outpatient care only	1.11	1.03	1.20	0.007
	Inpatient care only	4.96	4.06	6.05	< 0.001
	No care	25.30	23.30	27.50	< 0.001

**Fig. 2:**
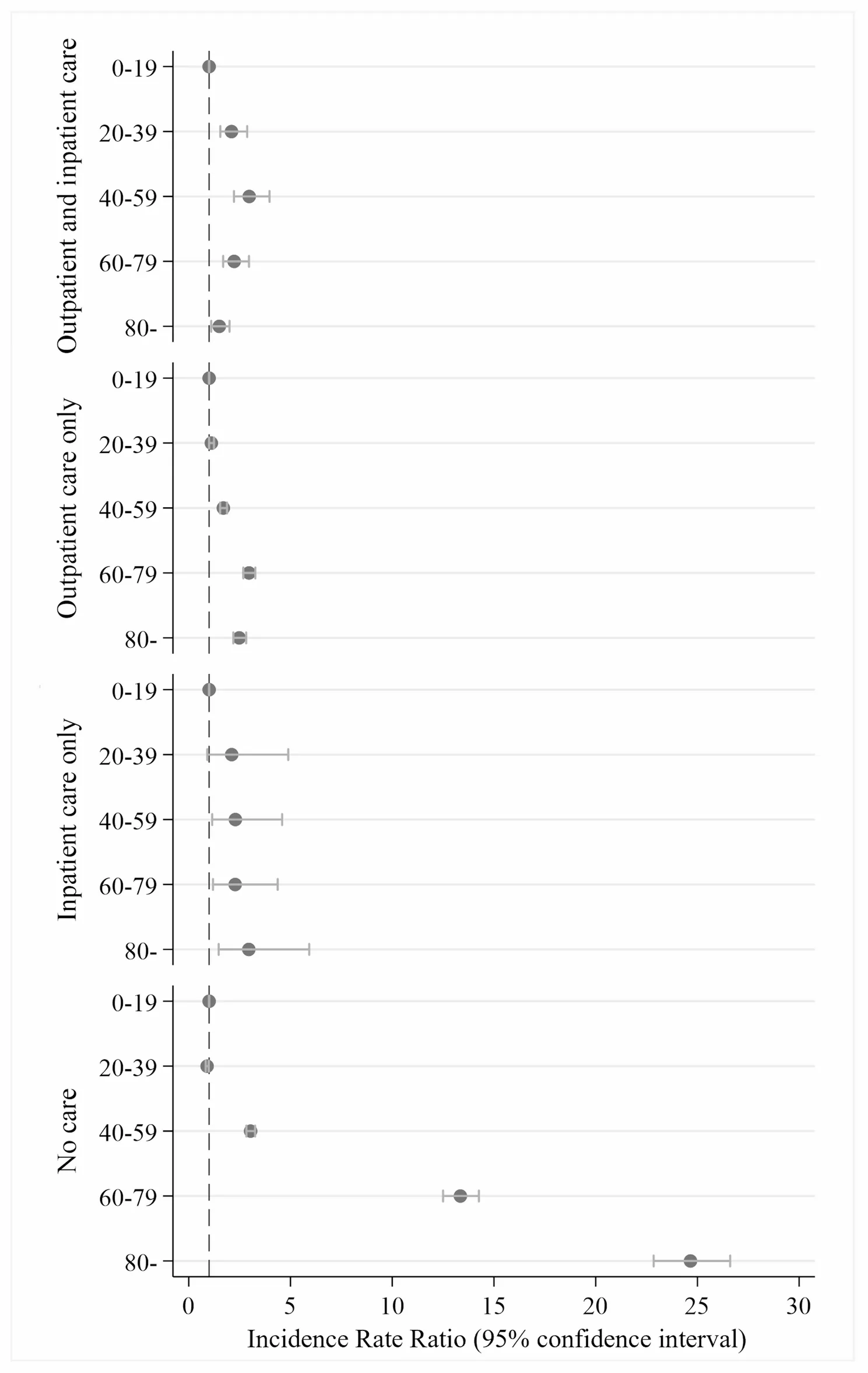
Subgroup analysis results of the General Linear Model (GLM) examining the association between receipt of public assistance and age group among participants diagnosed/confirmed as COVID-19. Footnote: The reference group comprised individuals aged 0–19 years. Lack of outpatient and inpatient care use in 2019 (no care) was the strongest risk factor for receiving public assistance among older participants

**Fig. 3:**
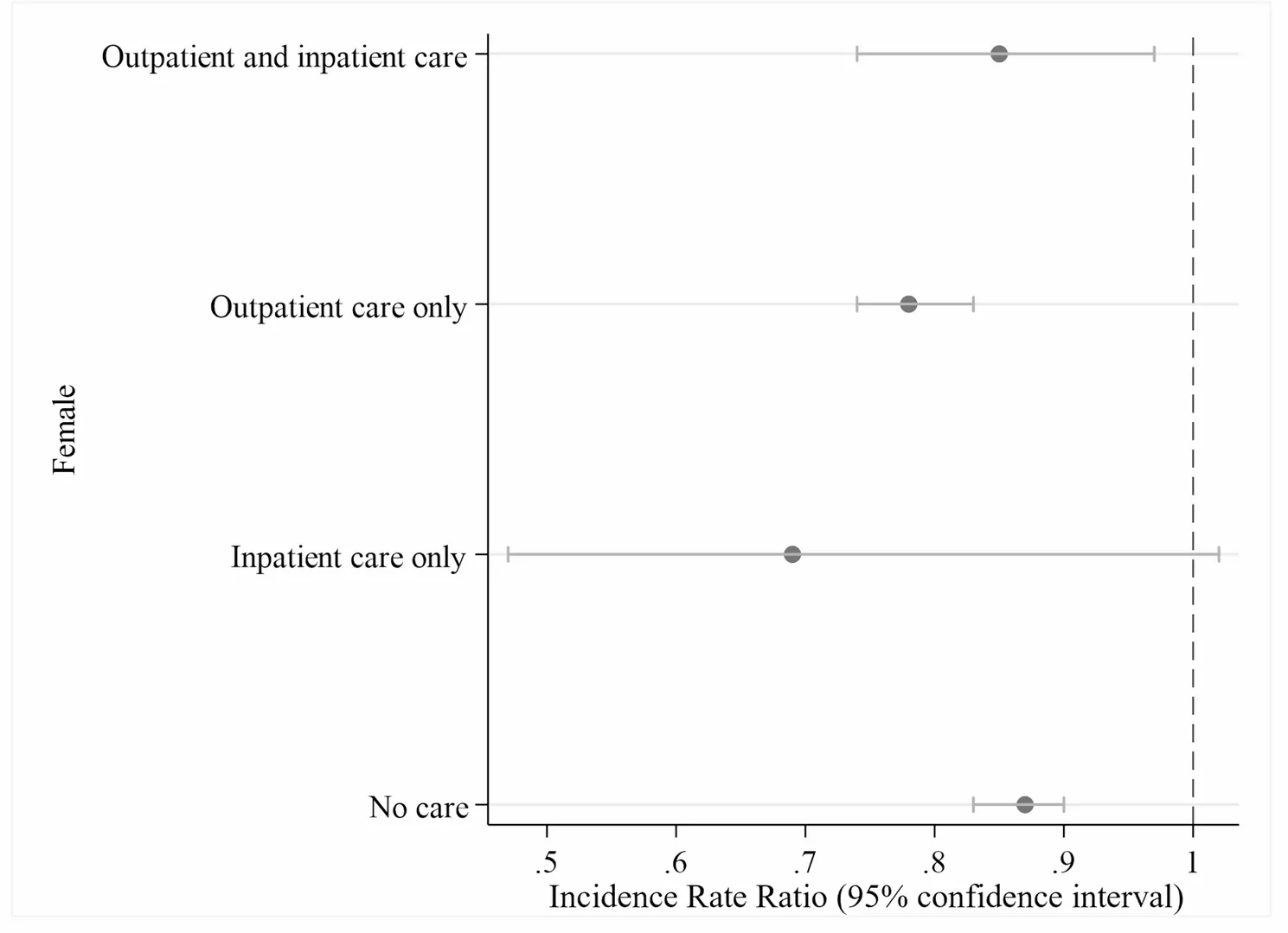
Subgroup analysis results of General Linear Model (GLM) examining the association between receipt of public assistance and sex among participants diagnosed/confirmed as COVID-19. Footnote: The incidence rate ratio was for women, with male as the reference group. Female participants generally had a lower risk of receiving public assistance compared with males, except among those who used inpatient care only

After we assessed the statistical significance of interaction terms (Supplementary Table 2), Figs. 2 and 3 present the results of subgroup analyses stratified by age group and sex, respectively. Lack of outpatient and inpatient care use in 2019 was the strongest risk factor for receiving public assistance among older participants (Fig. 2). Female participants generally had a lower risk of receiving public assistance compared with males, except among those who used inpatient care only (Fig. 3).

## Discussion

This study demonstrated that diagnosed/confirmed COVID-19 infection was associated with an increased likelihood of receiving public assistance among Japanese residents. Moreover, older individuals who had not used any hospital or clinic services in 2019 exhibited the highest risk of becoming WRs compared with other participants.

COVID-19 infection, along with the broader social conditions during the pandemic, appeared to increase the risk of requiring public assistance. During the observation period, the Japanese government covered the costs of diagnosed/confirmed COVID-19 treatment; therefore, infection did not result in out-of-pocket copayments. Other factors likely contributed to the observed increase in public assistance after diagnosed/confirmed COVID-19 infection. Previous studies have reported that socioeconomic disadvantage elevates the risk of COVID-19 infection [15, 16]. In addition, individuals infected with COVID-19 have been shown to experience a short-term reduction in household income [17]. Worsening health and economic conditions may reinforce one another, creating a cycle that further increases the risk of requiring public assistance.

Our subgroup analysis revealed a notable increase in the probability of welfare receipt immediately following a COVID-19 diagnosis among both male and female participants in the 0–19 years age group. Although minors are generally not independent earners, a diagnosis within this age group is likely to serve as a proxy for a household-level shock. Specifically, the infection of a child may lead to subsequent infection of guardians or require parents to take unpaid leave for caregiving, thereby depleting the household’s financial reserves and necessitating public assistance. This risk decreased over a relatively short period (within six months). This immediate but transient risk in the youngest cohort underscores how acute health shocks to any family member can compromise the economic stability of the entire household. In contrast, the risk of receiving public assistance remained elevated for a longer duration among working-age participants (20–39 and 40–59 years), remaining statistically significant for more than one year after infection. The persistent risk of welfare receipt observed in the 20–59 years age group may be partially attributed to long COVID. Symptoms, such as profound fatigue and cognitive impairment, can result in long-term work disability in the working-age population, leading to sustained reliance on the social safety net [18, 19]. This difference may be explained by several factors in addition to the potential effects of long COVID. One possible factor is generational differences in the proportion of regular versus non-regular employment. Regular employees are guaranteed salaries during leave, whereas most non-regular employees are not. According to long-term labor statistics, 48% of workers aged 15–24 were non-regular employees, compared with 28% of workers aged 25–54 [20, 21]. Among younger individuals, who have a higher proportion of non-regular employment, diagnosed/confirmed COVID-19 infection may have immediately compromised their financial stability, prompting them to seek public assistance. Additionally, most unemployed children depend on their parents’ incomes for their livelihood. In Japan, the average maternal age at childbirth in 2020 was 30.9 years [21]. This indicates that children of parents in the 20–39 years age group are generally infants or students who are financially dependent and require intensive care. This demographic structure is crucial for understanding the socio-economic impact of COVID-19 infection. When a child or parent in this age group is diagnosed with COVID-19, the necessity of caregiving and mandatory quarantine for close contacts leads parents to take leave from work. For those in non-regular employment without access to paid sick leave, this sudden loss of income, combined with limited financial reserves, can rapidly trigger a transition to public assistance.

We clarified that the risk of receiving public assistance varied according to the type of oral medication and that diagnosed/confirmed COVID-19 patients who had not used medical care before the pandemic experienced a significant age-related increase in the risk of receiving public assistance. This dataset did not include reliable diagnostic information for each participant [22, 23]. Therefore, the medications taken by participants could not be used to definitively determine their underlying medical conditions. In countries such as Japan, medical records cannot be shared across institutions, which makes it difficult to confirm a patient’s prior medical history during an initial consultation [24]. Consequently, patients may receive treatment for COVID-19 without a verified medical history. In such cases, reviewing a patient’s medication list can serve as a useful indicator for estimating their risk of becoming WRs. Furthermore, the high welfare receipt risk in the ‘no care’ group warrants a multi-faceted interpretation. From a disaster epidemiology perspective, natural disasters in Japan have been shown to increase risks for high-cost inpatient and institutional care; similarly, regarding COVID-19 as an acute health disaster, this group likely required public assistance due to sudden high-cost care burdens [25, 26]. Alternatively, this heterogeneous group reflects several structural factors. First, geographic misclassification is possible because study areas were defined by medical institution locations, not residential addresses. Second, a healthy-user effect likely existed among healthier younger individuals who had no prior clinical needs but lacked financial resilience against health shocks [27]. Third, the lack of claims may signify structural barriers, under-registration, or language barriers among marginalized populations, such as foreign or low-income workers lacking a stable ‘medical home’ in Japan. Additionally, female participants had a consistently lower risk of receiving public assistance compared with males, which was observed across all analyses.

This study, for the first time, revealed the risk of requiring public assistance in Japan after diagnosed/confirmed COVID-19 infection by using individual-level medical claims data directly, in addition to accounting for the broader impact of the COVID-19 pandemic. Although previous studies have examined the risk of COVID-19 infection and the challenges of implementing preventive measures among socially vulnerable groups—such as economically disadvantaged individuals and those experiencing homelessness—no prior studies have directly investigated the relationship between diagnosed/confirmed COVID-19 infection and subsequent WR [28–31]. Public assistance is provided based on objective indicators derived from local government surveys, rather than self-reported data from questionnaires. Using comprehensive data, we could estimate the risk of requiring public assistance. Furthermore, we evaluated the types of oral medications and prior medical institution use as clinically relevant information for the management of patients with COVID-19. Although insurance claims data such as the NDB have inherent limitations regarding clinical detail and patient background information, the identification of indicators that can be easily obtained through medical interviews adds clinical value and enhances the social epidemiological significance of these findings [32].

This study has several limitations. First, because welfare status is identified through medical claims, recipients who did not visit medical institutions were uncaptured. This outcome misclassification is likely non-differential and would bias estimates toward the null, indicating that our reported IRRs are conservative. Second, the database lacks detailed information on deaths, clinical diagnoses, long-term care services, and direct socioeconomic indicators (e.g., household income, education, and occupation). This precludes complete adjustment for baseline status, raising possibilities of comorbidity misclassification and residual confounding. Although we used 2019 healthcare utilization as a proxy for preexisting vulnerability, findings demonstrate a longitudinal association rather than a direct causal effect. Third, exposure classification was limited to diagnosed COVID-19 cases. Given early pandemic testing constraints, undiagnosed asymptomatic or mild cases were likely misclassified into the uninfected reference group. This non-differential exposure misclassification also biases estimate toward the null, further rendering our IRRs conservative. Fourth, monthly claims data introduce temporal ambiguity within the same calendar month, precluding definitive proof that the COVID-19 diagnosis preceded public assistance initiation. While our longitudinal design and pre-pandemic healthcare utilization adjustment mitigate reverse causality, residual confounding cannot be excluded. Finally, our analysis did not account for price elasticity. Price elasticity may influence the use of medical institutions by elderly individuals with high medical needs who have been receiving long-term welfare benefits since COVID-19 infection. Nevertheless, because these findings reflect changes under the national insurance system, we evaluated the overall effect of poverty among people infected with COVID-19.

The relationship between acute health shock and economic dependency is embedded in complex socioeconomic contexts. This transition is likely influenced by long-standing systemic structural biases and the accumulated impact of various social determinants of health. Our findings highlight the role of COVID-19 as a significant stressor within this intricate system; however, future studies utilizing more comprehensive multi-sectoral datasets, and advanced causal inference methodologies are needed to further disentangle the acute effects of the pandemic from chronic socioeconomic vulnerabilities.

## Conclusions

This study is the first to longitudinally track individuals infected with COVID-19 and examine their association with subsequent WR. The timing and extent of public assistance needs varied by age and sex, suggesting that future pandemic and disaster-related support policies should incorporate these demographic factors. In addition, we found that older individuals who did not utilize medical facilities before the pandemic were significantly more likely to require public assistance following infection. Further research is warranted to clarify why these individuals do not access medical care and to determine whether regular medical engagement could mitigate this risk. 

## Supplementary Information

Supplementary Material 1.

## Data Availability

The data that support the findings of this study are available from the Ministry of Health, Labour and Welfare, but restrictions apply to the availability of these data, which were used under license for the current study, and so are not publicly available. However, data are available from the authors upon reasonable request and with permission from the Ministry of Health, Labour and Welfare.

## Abbreviations

GEEs: Generalized Estimating Equations
GLM: General Linear Model
IRR: Incidence Rate Ratio
WRs: Welfare recipients
NDB: National Database of Health Insurance Claims
