# Supplementary material for: Association of COVID-19 infection with welfare recipients in Japan: a retrospective cohort study
Source: BMC Prim Care. 2026 Jul 31;27:390. doi: 10.1186/s12875-026-03504-1 (PMC13715272; doi:10.1186/s12875-026-03504-1)
Supplement: Supplementary file 1 [file 12875_2026_3504_MOESM1_ESM.docx]

Supplementary Table 1. Results of generalized estimating equations examining the association between receipt of public assistance and diagnosed/confirmed COVID-19 status including interaction terms

|  | | IRR | 95% CI | | P value |
| --- | --- | --- | --- | --- | --- |
|  |  |  | Under | Upper |  |
| Age category | 0–19 | Reference | | | |
|  | 20–39 | 1.40 | 1.34 | 1.47 | <0.001 |
|  | 40–59 | 2.66 | 2.55 | 2.78 | <0.001 |
|  | 60–79 | 4.45 | 4.26 | 4.64 | <0.001 |
|  | ≥80 | 4.85 | 4.64 | 5.07 | <0.001 |
| Sex | Male | Reference | | | |
|  | Female | 0.85 | 0.83 | 0.87 | <0.001 |
| Diagnosed/confirmed COVID-19 status | Uninfected | Reference | | | |
|  | Month of infection | 3.28 | 3.18 | 3.39 | <0.001 |
|  | 1–3 months after infection | 1.21 | 1.17 | 1.25 | <0.001 |
|  | 4–6 months after infection | 0.99 | 0.94 | 1.04 | 0.635 |
|  | 7–9 months after infection | 0.87 | 0.8 | 0.95 | 0.001 |
|  | 10–12 months after infection | 0.78 | 0.67 | 0.9 | 0.001 |
|  | >1 year after infection | 0.72 | 0.57 | 0.9 | 0.005 |
| Calendar Quarter | 2020 Q1 | Reference | | | |
|  | 2020 Q 2 | 1.70 | 1.67 | 1.72 | <0.001 |
|  | 2020 Q 3 | 2.41 | 2.37 | 2.44 | <0.001 |
|  | 2020 Q 4 | 3.02 | 2.97 | 3.07 | <0.001 |
|  | 2021 Q 1 | 3.42 | 3.36 | 3.48 | <0.001 |
|  | 2021 Q 2 | 3.97 | 3.9 | 4.04 | <0.001 |
|  | 2021 Q 3 | 4.35 | 4.28 | 4.43 | <0.001 |
|  | 2021 Q 4 | 4.90 | 4.82 | 4.99 | <0.001 |
|  | 2022 Q 1 | 4.94 | 4.86 | 5.03 | <0.001 |
|  | 2022 Q 2 | 5.40 | 5.3 | 5.49 | <0.001 |
|  | 2022 Q 3 | 5.45 | 5.36 | 5.55 | <0.001 |
|  | 2022 Q 4 | 5.73 | 5.63 | 5.83 | <0.001 |
| Interaction term between age category and diagnosed/confirmed COVID-19 status | | | | | |
| 20–39 | Month of infection | 0.79 | 0.76 | 0.83 | <0.001 |
|  | 1–3 months after infection | 1.11 | 1.06 | 1.16 | <0.001 |
|  | 4–6 months after infection | 1.15 | 1.08 | 1.23 | <0.001 |
|  | 7–9 months after infection | 1.23 | 1.12 | 1.36 | <0.001 |
|  | 10–12 months after infection | 1.33 | 1.13 | 1.58 | 0.001 |
|  | >1 year after infection | 1.31 | 1.02 | 1.68 | 0.033 |
| 40–59 | Month of infection | 0.76 | 0.74 | 0.79 | <0.001 |
|  | 1–3 months after infection | 1.30 | 1.25 | 1.36 | <0.001 |
|  | 4–6 months after infection | 1.35 | 1.28 | 1.43 | <0.001 |
|  | 7–9 months after infection | 1.42 | 1.3 | 1.55 | <0.001 |
|  | 10–12 months after infection | 1.52 | 1.29 | 1.78 | <0.001 |
|  | >1 year after infection | 1.53 | 1.21 | 1.95 | <0.001 |
| 60–79 | Month of infection | 0.82 | 0.79 | 0.85 | <0.001 |
|  | 1–3 months after infection | 1.47 | 1.42 | 1.53 | <0.001 |
|  | 4–6 months after infection | 1.48 | 1.4 | 1.57 | <0.001 |
|  | 7–9 months after infection | 1.51 | 1.38 | 1.65 | <0.001 |
|  | 10–12 months after infection | 1.54 | 1.32 | 1.8 | <0.001 |
|  | >1 year after infection | 1.48 | 1.18 | 1.88 | 0.001 |
| ≥80 | Month of infection | 0.68 | 0.66 | 0.71 | <0.001 |
|  | 1–3 months after infection | 1.24 | 1.19 | 1.3 | <0.001 |
|  | 4–6 months after infection | 1.19 | 1.12 | 1.26 | <0.001 |
|  | 7–9 months after infection | 1.19 | 1.08 | 1.3 | <0.001 |
|  | 10–12 months after infection | 1.18 | 1.01 | 1.39 | 0.04 |
|  | >1 year after infection | 1.13 | 0.89 | 1.43 | 0.32 |
| Interaction term between sex and diagnosed/confirmed COVID-19 status | | | | | |
| Female | Month of infection | 0.94 | 0.93 | 0.96 | <0.001 |
|  | 1–3 months after infection | 0.96 | 0.95 | 0.98 | 0.001 |
|  | 4–6 months after infection | 1.04 | 1.01 | 1.07 | 0.003 |
|  | 7–9 months after infection | 1.08 | 1.04 | 1.12 | <0.001 |
|  | 10–12 months after infection | 1.12 | 1.07 | 1.18 | <0.001 |
|  | >1 year after infection | 1.15 | 1.08 | 1.22 | <0.001 |

Footnote:

Abbreviations: IRR, incidence rate ratio; CI, confidence interval.

Supplementary Table 2. Results of General Linear Model examining the association between receipt of public assistance including interaction terms

| Variable | | IRR | 95% CI | | P value |
| --- | --- | --- | --- | --- | --- |
|  |  |  | Under | Upper |  |
| Age category | 0–19 | Reference | | | |
|  | 20–39 | 0.90 | 0.83 | 0.98 | 0.017 |
|  | 40–59 | 3.05 | 2.84 | 3.27 | <0.001 |
|  | 60–79 | 13.57 | 12.72 | 14.48 | <0.001 |
|  | ≥80 | 25.19 | 23.38 | 27.13 | <0.001 |
| Sex, n (%) | Male | Reference | | | |
|  | Female | 0.87 | 0.84 | 0.91 | <0.001 |
| Prescription | Hypnotic and sedative agents, antidepressants | 1.56 | 1.45 | 1.69 | <0.001 |
|  | Psychotropic agents | 2.29 | 2.12 | 2.47 | <0.001 |
|  | Antihypertensive agents | 1.14 | 1.06 | 1.23 | 0.001 |
|  | Lipid-lowering agents | 0.79 | 0.72 | 0.85 | <0.001 |
|  | Antidiabetic agents | 1.32 | 1.20 | 1.46 | 0.002 |
|  | Chinese herbal medicines | 0.75 | 0.70 | 0.81 | <0.001 |
| Utilization of care services in 2019 | No care | Reference | | | |
|  | Outpatient care only | 0.15 | 0.11 | 0.19 | <0.001 |
|  | Inpatient care only | 0.12 | 0.11 | 0.13 | <0.001 |
|  | Outpatient and inpatient care | 0.73 | 0.41 | 1.32 | 0.303 |
| Interaction term between age category and utilization of care services in 2019 | | | | | |
| 20–39 | Outpatient care only | 2.27 | 1.64 | 3.13 | <0.001 |
| 40–59 | Inpatient care only | 1.22 | 1.07 | 1.40 | 0.004 |
| 60–79 | Outpatient and inpatient care | 2.35 | 1.01 | 5.44 | 0.047 |
| ≥80 | Outpatient care only | 0.88 | 0.66 | 1.19 | 0.41 |
| 20–39 | Inpatient care only | 0.55 | 0.49 | 0.62 | <0.001 |
| 40–59 | Outpatient and inpatient care | 0.76 | 0.38 | 1.53 | 0.44 |
| 60–79 | Outpatient care only | 0.15 | 0.11 | 0.20 | <0.001 |
| ≥80 | Inpatient care only | 0.21 | 0.18 | 0.23 | <0.001 |
| 20–39 | Outpatient and inpatient care | 0.18 | 0.09 | 0.34 | <0.001 |
| 40–59 | Outpatient care only | 0.05 | 0.04 | 0.07 | <0.001 |
| 60–79 | Inpatient care only | 0.09 | 0.08 | 0.11 | <0.001 |
| ≥80 | Outpatient and inpatient care | 0.13 | 0.06 | 0.26 | <0.001 |
| Interaction term between sex and utilization of care services in 2019 | | | | | |
| Female | Outpatient care only | 0.96 | 0.83 | 1.10 | 0.53 |
|  | Inpatient care only | 0.90 | 0.84 | 0.97 | 0.005 |
|  | Outpatient and inpatient care | 0.79 | 0.53 | 1.18 | 0.24 |

Footnote:

Abbreviations: IRR, incidence rate ratio; CI, confidence interval.
